# A new species of the genus *Arhynchite* (Annelida, Echiura) from sandy flats of Japan, previously referred to as *Thalassema owstoni* Ikeda, 1904

**DOI:** 10.3897/zookeys.312.5456

**Published:** 2013-06-24

**Authors:** Masaatsu Tanaka, Teruaki Nishikawa

**Affiliations:** 1 Department of Biology, Faculty of Science, Toho University, 2-2-1 Miyama, Funabashi, Chiba 274-8510, Japan

**Keywords:** Annelida, Echiura, new species, *Arhynchite hayaoi*, *Thalassema owstoni*, sandy flat, Seto Inland Sea

## Abstract

A new echiuran, *Arhynchite hayaoi* **sp. n.**, is described from newly collected specimens from sandy flats of the Seto Inland Sea, Japan, together with many museum specimens, including those once identified as *Thalassema owstoni* Ikeda, 1904 or *Arhynchite arhynchite* (Ikeda, 1924). The new species is clearly distinguishable from its congeners by the smooth margin of gonostomal lips and lack of rectal caecum. Brief references are also made to the morphological distinction between the new species and *Thalassema owstoni*, originally described from the deep bottom on the Japanese Pacific coast.

## Introduction

Echiurans are a small group of marine coelomates, previously classified as a distinct phylum, but now often included in the Phylum Annelida on the basis of recent molecular phylogenetic analyses (McHugh 1997, Bleidorn et al. 2003, Struck et al. 2007, 2011). The Japanese echiuran fauna was extensively studied by Prof. Iwaji Ikeda and Dr Hayao Sato in the early and middle parts of the last century, respectively, resulting in our present understanding of the known fauna consisting of ca 20 species (Nishikawa 1992). However, many taxonomic problems remain unsolved, one of which concerns an echiuran called “kouju” in Japanese (probably meaning “good bait”). This echiuran was once abundant and collected in great numbers from intertidal or subtidal sandy bottoms for fish bait in the Seto Inland Sea, Japan (Mori et al. 1932, Ishikawa 1938); however, its density has lately declined too greatly for such use (Nishikawa 2007, 2012, Saito et al. 2011).

The echiuran in the present study was first identified as *Thalassema mucosum* (currently accepted as *Anelassorhynchus mucosus* (Ikeda, 1904)) by Mori et al. (1932). It was soon referred to by Sato (1934), one of the above-mentioned specialists, as *Thalassema owstoni* Ikeda, 1904, established for a damaged deep-water Japanese specimen, after his own examination of specimens newly collected from Onomichi Bay in the Seto Inland Sea. Although Sato’s (1934) description lacked any information on their internal morphology or the reason for his corrected identification, his taxonomy has been followed by subsequent authors (Sato 1935, 1939, Ishikawa 1938, Stephen and Edmonds 1972, Nishikawa 1992, 2012).

We have compared echiurans newly collected from a sandy flat near Onomichi Bay with the specimens deposited in the Tohoku University Museum (TUM), collected from the bay in 1931 and labeled as “*Thalassema owstoni* Ikeda” and “kouji” (probably equivalent to “kouju”), probably by Sato, who then belonged to the Tohoku Imperial University, the predecessor of Tohoku University. We found the old and new specimens to be very similar. Further, we found a significant difference between the specimens and the description of *Thalassema owstoni* Ikeda, 1904, although we attempted to examine its holotype and collect new specimens from the type locality, we have so far been unsuccessful. Thus, we herein describe “kouju” as a new species of the genus *Arhynchite* Satô, 1937.

## Materials and methods

We collected new specimens from a sandy flat, named Hachi-no-higata, at the mouth of the Kamogawa River, Takehara, Hiroshima Prefecture, Japan in 2011. The specimens were relaxed with menthol, and minute pieces of proboscis were taken from the holotype and two paratypes and fixed with pure ethanol for future molecular analyses; all remaining material was fixed in 10% seawater formalin and then stored in 70% ethanol. Further materials have been retained at our Laboratory of Taxonomy, Toho University (LTTU), the Department of Zoology, University Museum, University of Tokyo (UMUTZ), or TUM. The type series, as well as the two non-type ones, are deposited in the National Museum of Nature and Science, Tsukuba, Japan (NSMT).

Observations, dissections, and drawings were made under a stereoscopic microscope. Trunk length (TL) and proboscis length (PL) were measured. General terminology was based on Stephen and Edmonds (1972) and Nishikawa (2004).

## Taxonomy

### Arhynchite hayaoi sp. n.

urn:lsid:zoobank.org:act:598901D0-6F77-4F4B-AA50-343FEAD4F9E5

http://species-id.net/wiki/Arhynchite_hayaoi

**Figure 1.**
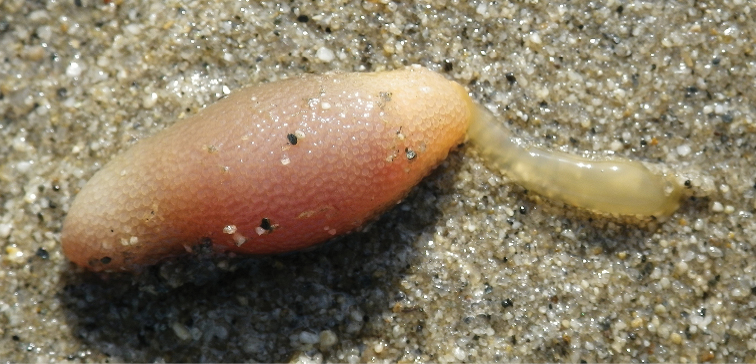
*Arhynchite hayaoi* sp. n., found at the type locality, with the proboscis extending toward the right.

**Figure 2.**
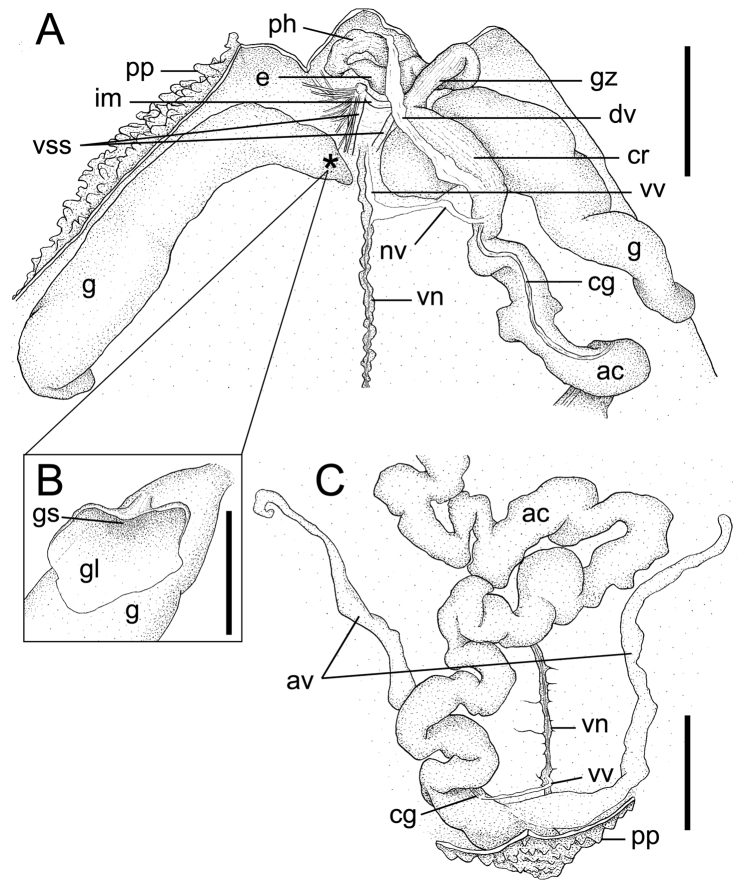
*Arhynchite hayaoi* sp. n., paratype (NSMT-Ec 104). **A** internal morphology of the anterior end of the trunk, dorsal view; an asterisk indicates the position of the gonostome **B** gonostome and gonostomal lip, magnified **C** internal morphology of the posterior end of the trunk, dorsal view. Abbreviations: **ac** alimentary canal; **av** anal vesicle; **cg** ciliated groove; **cr** crop; **dv** dorsal vessel; **e** esophagus; **g** gonoduct; **gl** gonostomal lip; **gs** gonostome; **gz** gizzard; **im** interbasal muscle; **nv** neurointestinal vessel; **ph** pharynx; **pp** papillae; **vn** ventral nerve cord; **vss** ventral setal sac; **vv** ventral vessel. Scales: **A** and **C** 5 mm; **B** 1 mm.

New Japanese name: Setouchi-dochikuchi-yumushi Figs 1 ,2

Thalassema owstoni : Sato 1934, p. 249, figs 2 and 3; Satô 1939, p. 354.
Arhynchite arhynchite : Nishikawa 2001, pp. 137–138.

#### Specimens examined.

**Holotype:** NSMT-Ec 100, mature male, TL 25 mm, PL>5 mm (proboscis damaged), Hachi-no-higata sandy flat at the mouth of the Kamogawa River, Hiroshima Pref., Japan (34°19.42'N, 132°53.88'E), May 18, 2011, collected by M. Tanaka and T. Nishikawa. **Paratypes:** NSMT-Ec 101–105, 3 males + 2 females, TL 28–40 mm, PL 6 mm, collection data as for the holotype. **Non-type specimens:** TUM Echiurida 2-11, labeled as “*Thalassema owstoni* Ikeda” probably by H. Sato, 8 males + 6 females + 1 specimen of unknown sex, TL 29–73 mm, proboscis absent, Onomichi Bay, Hiroshima Pref., Japan (34°24'N, 133°12'E), March 7, 1931, collected by Takashi Gamo; TUM Echiurida 1-5, labeled as “*Thalassema owstoni* Ikeda” and “kouji” probably by H. Sato, 2 males + 3 females + 1 of unknown sex, TL 31–62 mm, PL 16–33 mm, Onomichi Bay, Hiroshima Pref., Japan (34°24'N, 133°12'E), March 7, 1931, by Takashi Gamo; NSMT-Ec 106, 1 of unknown sex, TL 28 mm, proboscis absent, intertidal sandy flat at the mouth of the Kamogawa River, Hiroshima Pref., Japan (34°19.42'N, 132°53.88'E), July 8, 2006, by Masanori Sato; NSMT-Ec 107, 1 of unknown sex, TL 33 mm, proboscis absent, intertidal sandy to muddy flat on Ikarise Islet, located in a channel of Lake Hamana, Shizuoka Pref., Japan (34°41.08'N, 137°35.98'E), April 19, 2003, by Shoichi Kimura; UMUTZ-Ecur-2, 1 of unknown sex, TL 55 mm, proboscis absent, above low-water mark, Tomono-ura, Hiroshima Pref., Japan (34°22'N, 133°23'E), July 1882, by I. Ikeda, reported as *Arhynchite arhynchite* (Ikeda) by Nishikawa (2001); UMUTZ-Ecur-10, 1 of unknown sex, TL 38 mm, proboscis absent, Misaki, Sagami Bay, Kanagawa Pref., Japan (35°09’N, 139°36’E), collection data unknown, reported as *Arhynchite arhynchite* by Nishikawa (2001). **Specimens for comparison:** *Arhynchite arhynchite*; LTTU-Y009, 1 female + 3 of unknown sex, TL 42–74 mm, off Abashiri, Hokkaido, Japan, 7–12 m depth, September 17, 2001, collected by Yasuhiro Kuwahara.

#### Diagnosis.

Trunk up to 80 mm long in preserved specimens. Leaf-like gonostomal lips with smooth margins. Neurointestinal vessel unbifurcated. Ring vessel absent. Rectal caecum absent. Anal vesicles fastened basally to the trunk wall by mesenteries.

#### Description.

In life, trunk colored pinkish yellow and proboscis pale yellow (Fig. 1). Coloration fading to pale white or beige after fixation with formalin.

In preserved specimens, TL ranging from 25 to 73 mm (n = 28) and PL ranging from 6 to 33 mm (n = 8). Proboscis, often detached from trunk, elongated and slightly expanded at its extremity, with its proximal end forming small lower cup around mouth. Trunk wall thickened and covered with numerous distinct papillae, especially prominent (up to 1 mm) at both extremities. Trunk musculature consisting of outermost circular, middle longitudinal, and innermost oblique layers, all of which continuous throughout.

Paired ventral setae of usual form, with strong interbasal muscle (Fig. 2A), but only very rarely single seta without interbasal muscle in 2 of 6 non-type specimens in TUM Echiurida 1-5.

Paired gonoducts situated slightly behind ventral setae (Fig. 2A). Gonostomes situated proximally, each with leaf-like gonostomal lip with smooth margin (Fig. 2B); lip plicated marginally, probably due to fixation in many non-type specimens (TUM Echiurida 1-5, 2-11).

Long and convoluted alimentary canal, filled with sand grains and elliptical fecal pellets, ca 2 mm in long axis (Fig. 2A, C). Anterior part of alimentary canal, so-called foregut, divided into pharynx, esophagus, gizzard, and crop (Fig. 2A). Intestine following foregut fastened to trunk wall with numerous thread-like mesenteries and divided into pre-siphonal, siphonal, and post-siphonal parts (Fig. 2A, C). Pre- and post-siphonal parts having a ciliated groove; elongated pre-siphonal part about twice as long as TL (Fig. 2A). Rectal caecum absent (Fig. 2C).

Vascular system composed of dorsal, neurointestinal, and ventral vessels, without ring vessel (Fig. 2A). Dorsal vessel attached to entire length of crop (Fig. 2A). Ventral vessel running along almost entire length of ventral nerve cord and terminating at posterior end of post-siphonal intestine near anus (Fig. 2A, C). Neurointestinal vessel, although injured in holotype, issuing from ventral vessel slightly behind gonostomal level and terminating at anterior end of intestine without bifurcation (Fig. 2A). In 6 non-type specimens (5 in TUM Echiurida 2-11 and 1 in TUM Echiurida 1-5), ventral vessel issuing additional branch forward at ventral origin of neurointestinal vessel, with the branch terminally forming small loop around interbasal muscle. Further, in another non-type specimen of TUM Echiurida 1-5, origin of neurointestinal vessel shifted far forward, crossing interbasal muscle but without additional branches.

Paired simple anal vesicles, ca one-third of TL in holotype, while rarely attaining TL in other specimens, covered wholly with numerous microscopic ciliated funnels and fastened basally to trunk wall by some mesenteries (Fig. 2C).

#### Etymology.

The specific name is dedicated to the late Dr Hayao Sato who made a significant contribution to the taxonomy of echiurans, sipunculans, and priapulids in Japan and adjacent waters.

#### Distribution.

Hiroshima Pref. (Seto Inland Sea), Ikarise Islet at the entrance of Lake Hamana, and Misaki (Sagami Bay), Japan, intertidal to subtidal, sandy to muddy bottoms.

#### Remarks.

Table 1 gives a comparison of all known species and the newly described species assigned to the genus *Arhynchite*. *Arhynchite hayaoi* sp. n. is distinguishable from *Arhynchite arhynchite*, the only congener recorded to date from Japanese coasts, by the absence of such digitiform projections or irregular serrations along the margin of the gonostomal lips as are present in *Arhynchite californicus* and *Arhynchite pugettensis*, respectively (Fisher 1949, pls. 30, 32), and by the complete lack of bifurcation in the neurointestinal vessel.

**Table 1.** A comparison of all known species referred to the genus *Arhynchite*. <br/>

**Species**	**Rectal caecum**	**Ring vessel**	**Bifurcation of neurointestinal vessel**	**Margin of gonostomal lip**	**Mesenteries mainly fastened to**	**Type locality**	**Sources**
*Arhynchite hayaoi* sp. n.	absent	absent	absent	smooth	body wall	Seto Inland Sea, Japan	This study
*Arhynchite arhynchite*	absent	absent	barely detectable	with digitiform projections	body wall	Probably around Hokkaido, Japan	Ikeda (1924), Satô (1937), This study
*Arhynchite californicus*	absent	absent	absent	with digitiform projections	body wall	Monterey Bay, California, USA	Fisher (1949)
*Arhynchite hiscocki*	absent	absent	absent	with digitiform projections	intestine	Dunwich, Queensland, Australia	Edmonds (1960, 1987)
*Arhynchite inamoenus*	absent	absent	absent	with irregular serrations	unknown	Monterey Bay, California, USA	Fisher (1946)
*Arhynchite pugettensis*	absent	present	absent	with irregular serrations	body wall	Puget Sound, Washington, USA	Fisher (1949)
*Arhynchite paulensis*	present	absent	unknown	with irregular serrations	intestine	Araça Beach, São Paulo, Brazil	Amor (1971)
*Arhynchite rugosus*	present	unknown	unknown	smooth	unknown	Jiaozhou Bay, Shandong, China	Chen and Yeh (1958)

Nishikawa (2001) recorded *Arhynchite arhynchite* from Sagami Bay and the Seto Inland Sea. Our detailed re-examination of these specimens revealed that they are assignable to *Arhynchite hayaoi*, instead of *Arhynchite arhynchite*. Thus, it is possible that *Arhynchite hayaoi* is widely distributed in the Seto Inland Sea and the Pacific coasts of Middle Japan, whereas *Arhynchite arhynchite*, with its type locality probably originating near Hokkaido (Ikeda 1924, Satô 1937), is restricted to cold northern waters.

## Discussion

*Thalassema owstoni* was established by Ikeda (1904, pp. 62–63, figs 18, 96–97) based solely on the holotype, dredged from 180 fathoms (ca 330 m) deep in the Uraga Channel at the entrance of Tokyo Bay. Since then, no further specimens have been collected. According to the original description, “The specimen [= the holotype] is torn near the posterior end of the body” (p. 62), probably lacking the greater part of the alimentary canal, the anal vesicles, and the vascular system. Our efforts to locate the holotype of *Thalassema owstoni* in order to gain more information have so far been unsuccessful. On the basis of the original description, however, this species is clearly distinguishable from *Arhynchite hayaoi* by the shape of the gonostomal lips (funnel-shaped in *Thalassema owstoni* vs. leaf-like in *Arhynchite hayaoi*).

Thus, the taxonomic identity of *Thalassema owstoni* remains unknown. Bock (1942) provisionally regarded this species as belonging to *Maxmuelleria* based on Ikeda’s original description, following Wharton’s key (1913, p. 261) that “Anal trees bear large funnels situated close together on long stalks.” This taxonomic treatment, however, is not followed by subsequent authors (Stephen and Edmonds 1972, Saxena 1984, Biseswar 1988, Nishikawa 1992, 2012). Without new material or the rediscovery of the holotype, the identity of *Thalassema owstoni* remains unresolved.

*Thalassema fuscum* Ikeda, 1904 is also similar to *Arhynchite hayaoi* in terms of living coloration (Ikeda 1904, p. 70 and fig. 21), the trunk surface densely covered with prominent papillae, and the occurrence of a single pair of gonoducts with the gonostomal lip expanded as a leaf. However, *Thalassema fuscum* is distinguished from *Arhynchite hayaoi* by the existence of a conspicuous ring vessel and a bifurcated neurointestinal vessel, as well as by the absence of interbasal muscle. Our present understanding of the last feature comes from the fact that *Thalassema fuscum*’s original description (Ikeda 1904, pp. 69–70) lacks any information on the muscle, but the taxonomic key of all species mentioned in Ikeda’s monograph (1904, p. 82) clearly shows “No interbasal muscle” for *Thalassema fuscum*.

## Supplementary Material

XML Treatment for Arhynchite hayaoi
